# Demographic factors affecting quality of life of hemodialysis patients – Lahore, Pakistan

**DOI:** 10.12669/pjms.305.5239

**Published:** 2014

**Authors:** Muhammad Anees, Muzammil Riaz Malik, Tanzeel Abbasi, Zeeshan Nasir, Yasir Hussain, Muhamamd Ibrahim

**Affiliations:** 1 Dr. Muhammad Anees, MBBS, FCPS (Nephrology), Assistant Professor of Nephrology, Visiting Consultant Nephrologist, Shalamar Hospital Lahore, Pakistan. King Edward Medical University, Lahore, Pakistan.; 2 Dr. Muzammil Riaz Malik, MBBS, Registrar Nephrology Department. King Edward Medical University, Lahore, Pakistan.; 3 Dr. Tanzeel Abbasi, MBBS, Postgraduate Trainee, Nephrology Department, King Edward Medical University, Lahore, Pakistan.; 4 Dr. Zeeshan Nasir, MBBS, Postgraduate Trainee, Nephrology Department, King Edward Medical University, Lahore, Pakistan.; 5 Dr. Yasir Hussain, MBBS, Postgraduate Trainee, Nephrology Department, King Edward Medical University, Lahore, Pakistan.; 6 Muhamamd Ibrahim, Associate Professor of Statistics, Registrar, Govt. M.A.O College, Lahore, Pakistan.

**Keywords:** ESRD, Hemodialyis, QOL, Demographic factors, WHO QOL BREF

## Abstract

***Objective:*** The objective of the study was to determine the demographic factors affecting Quality Of Life (QOL) of hemodialysis (HD) patients.

***Methods: ***This observational study was conducted at Shalamar Hospital, Lahore. Patients of End Stage Renal Disease (ESRD) and on maintenance HD for more than three months were included during the period March to June 2012. Patient of ESRD not on dialysis and Acute Renal Failure were excluded. One hundred and twenty five patients who fulfilled the criteria were included. Demographic data containing age, sex, residence, socio economic status, education, mode of traveling for dialysis, total time consumed in dialysis were collected by the investigators. QOL index was measured using 26 items, WHO QOL BREF.

***Results: ***There were 89(71.2%) male and 36(28.8%) female patients. Environmental domain score was highest (p=0.000) than all other domains in HD Patients. In overall analysis age, marital status and total time consumed in getting HD effect QOL significantly (p=<0.05). In domain wise analysis, male has better QOL in social relationship domain than female. Age has negative relationship with physical health and psychological health domain. QOL of unmarried and literate patients is significantly better (p=<0.05) in physical health domain. Employed patients have better QOL in physical, psychological and social relationship domain (p=<0.05) than unemployed patients. Patients of residence of rural areas have better QOL in physical and environment domain. Financial status of HD patients affect QOL in social domain. Distance covered to reach hospital effect QOL in psychological domain (p=<0.05). Patients traveling in private transport have better QOL in environmental domain (p=<0.05). Total time consumed in getting HD effect social relation in QOL (p=<0.05). According to linear regression model, marital status is positive predictor and unemployment is negative predictor of QOL in physical health domain. Age is negative predictor of QOL in psychological domain, monthly income is positive predictor of QOL in domain. Unemployment is positive predictor of QOL in social relation domain. Monthly income and place of residence is positive predictor of QOL in environment domain.

***Conclusion: ***Gender, age, marital status, unemployment, residence of rural area, economical status, distance covered to reach hospital, mode of transport, total time consumed in getting HD, effect QOL in HD patient. Education level is a positive factor for improving QOL of HD patients.

## INTRODUCTION

Quality Of Life is defined as “an individual’s perception of their position in life in the context of the culture and value system where they live, and in relation to their goals, expectations, standards and concerns”.^1^ Health-related quality of life (HRQOL) represents the “physical, psychological, and social domains of health that are influenced by a person’s experience, beliefs, expectations, and perceptions.^2^ Studies have documented that ESRD patients receiving dialysis treatment have a lower quality of life than the people in the general population.^3^ Various studies have shown that level of hemoglobin, socioeconomic status, literacy, dialysis program, ethnic groups, sex, mobility, comorbidities (e.g., diabetes), malnutrition, depression^4^ and unsuccessful previous renal transplant affect QOL of dialysis patients.^5^

Type of renal replacement therapy also affects QOL in these patients.^6^ In Pakistan, there is late referral to nephrologist,^7^ inadequate dialysis,^8^ high rate of depression,^9^ malnutrition & anemia,^10^ which affect QOL of these patients. In Pakistan, nephrology services are not up to mark due to limited numbers of nephrologists (eighty for population of 163million)^11^ and only 40% of the patients have an access to dialysis services.^12^ There is deficiency of the trained personals leading to poor quality of dialysis services. There is very limited data in this aspect in our dialysis patients, so this multicentric study was conducted to assess the QOL of HD patients and demographic factors affecting it.

## METHODS

This observational study was conducted at Shalamar Hospital, Lahore. Patient of ESRD, on maintenance HD treatment for more than months from three HD centres (Shalimar Hospital n_1_=48, Mayo Hospital n_2_=56 and Doctors Hospital & Medical Center n_3_=21) of Lahore, were included as study subjects who were able to understand, speak/read the local language. Patients of ESRD, on HD for less than three months duration and acute renal failure were excluded from the study. Demographic data was collected using pre-designed questionnaire. QOL index was measured using 26 items WHO, QOL BREF (Urdu version Khan MN et al).^13^

The WHO QOL BREF Contains 26 questions relating to physical health, psychological, social and environmental status of patients. Question 1 asks about individuals overall perception of QOL and Question 2 is about overall perception of health. Remaining 24 questions are divided in to four domains. Out of the four domains one is physical health and other are psychological, social and environmental domains. All domains have different raw score ranges, for uniformity all raw scores were transformed to 4 – 20 range according to WHO guidelines higher scores show a better QOL. All four domains were taken as dependent variables and other demographic factors were taken as independent variables.

Data analysis was carried out using SPSS V-17. Descriptive analysis was expressed in the form of percentages for qualitative variable and   used for quantitative variables. Pearson correlation coefficient was used to calculate the relationship between demographic factors and QOL index. Multiple Linear Regression model was used to assess the significant demographic factors as predictor of QOL. Backward elimination method was used to determine the strongest predictors for each domain. One way ANOVA was used to compare mean QOL of different domains. A P-value < 0.05 was considered as statistical significant.

## RESULTS

In this study one hundred and twenty five patients were included, among them there were 89(71.2%) male and 36(28.8%) female patients. The environment domain showed a significantly high QOL score in HD patients than other domains (p < 0.05). Overall there was no statistically significant difference in the QOL of male and female patients (p=0.421). However, male (12.65±4.26) has better QOL (p=0.047) in domain 3(Social Relationships) as compared to female (11.00±3.99). Age have negative relationship with physical and psychological health domain and effect on QOL in overall analysis. Marital status effect QOL significantly in overall analysis (p=.049). QOL in unmarried is better as compared to married which is statistically significant. Education level does not affect QOL of HD patients in overall analysis but literate patients have better QOL in Domain 1(Physical health) than Illiterate which is statistically significant(p=0.024). Employed patients have better QOL in Domain 1, 2 & 3 than unemployed HD patients. Place of residence affects QOL i.e. patients of rural areas in domain 1 (Physical health) has better QOL as compared to patients living in urban. Financial status of the patients effect QOL of HD patients. Distance covered to reach hospital does not effect QOL in multivariate analysis but in univariate analysis QOL effect significantly in psychological domains. Mode of transport effect QOL in domain wise analysis. Total time consumed in getting HD effect QOL in overall analysis. Family members of HD patients does not effect QOL in overall and domain wise analysis.

Multiple Linear Regression Models of QOL Index on various significant predictors for different domain are as follows.

***Estimated final regression model of various domain***

*Physical Health Domain*

*Y (QOL) = 10.839 + 2.581 X*_1_ *(Marital Status) - 2.108 X*_2_ *(Employment)*

*Psychological Domain*

*Y (QOL) = 13.633 - 0.055 X*_1_ *(Age) + 0.740 X*_2_ *(Monthly Income)*

*Social Relationship Domain*

*Y (QOL) = 15.908 - 1.5321 X*_1_ *(Employment)*

*Environment Domain*

*Y (QOL) = 10.546 + 0.718 X*_1_ *(Monthly Income) - 1.016 X*_2_ *(Place of Residence)*

**Table-I:** Demographic data of HD patients N=125

**Sr. No.**	**Variables**	**N (%)**		**Statistic**	**p-value**
1	Domain				
	Physical Health	125	10.3 + 3.48	F=14.32	0.001*
	Psychological	125	12.22 + 2.83		
	Social	125	12.10 + 4.06		
	Environment	125	12.86 + 2.33		
2	Gender				
	Male	89 (71.2)	48.02 + 10.15	t=0.807	0.421
	Female	36 (28.8)	46.44 + 9.25		
3	Age				
	> 45 Year	75 (60)	50.30 + 8.92	t=2.6454	0.011*
	< 45 Year	50 (40)	45.75 + 10.13		
4	Marital Status				
	Married	99 (79.2)	46.68 + 9.77	t=1.988	0.049*
	Unmarried	26 (28.8)	50.96 + 9.784		
5	Education				
	Literate	84 (67.2)	48.35 + 9.87	t=1.261	0.210
	Illiterate	41 (32.85)	45.98 + 9.84		
6	Employment				
	Unemployed	103 (82.4)	46.59 + 9.37	t=2.43	0.016*
	Employed	22 (17.6)	52.14 + 11.12		
7	Place of Residence				
	Urban	100 (80)	46.92 +9.66	t=-1.472	0.144
	Rural	25 (20)	50.10 + 10.43		
8	Monthly Income (Rs)				
	<10,000	76(60.8)	46.76 + 9.41	F=0.989	0.375
	10,000-30,000	33(26.4)	49.64 + 11.55		
	>30,000	16(12.8)	47.12 + 8.24		
9	Distance covered to reach hospital (Km)				
	> 5	94(75.2)	46.98 + 9.03	t=1.2130	0.248
	< 5	31(24.8)	9.35 + 10.588		
10	Mode of Transport				
	Private	50(40)	48.12 +11.12	F=0.332	0.718
	Public	60(48)	46.83 + 9.20		
	Others	15(12)	48.66 + 8.45		
11	Total Time consumed in getting HD (Hours)				
	> 6	76 (60.8)	48.74 +10.463	t=1.657	0.100
	< 6	49 (39.2)	45.76 + 8.721		
12	Family Members				
	> 8	38 (30.4)	47.92 + 10.05	t=0.263	0.793
	< 8	87 (69.6 )	47.41 + 9.87		

* Statistically Significant Value.

## DISCUSSION

ESRD is a chronic disease which causes a high level of hindrance in different aspects of the patient’s live, leading to poor QOL. Patients with low HRQOL pull out from HD treatment more commonly.^14^ In domain wise analysis of HD patients, there was highest score in environment domain and lowest in physical domain. Similar pattern was observed by Salim K et al.^15^ The reason for worst score in physical domain in HD patients is dependence on medical support, strict treatment regime of twice or thrice weekly HD, stat of pain and misery all the times, disturbed sleep and immobility which affect their QOL. These patients have to face many adversities e.g. specific dietary regimen, changes in their body image, dependence on machines which increases anxiety and affect QOL. Other factors are late referral to nephrologist, use of primary access catheter for dialysis and inadequate dialysis which affect QOL according to international literature.

Gender affects QOL in general population and HD patients as well.^15^^,^^16^ Females have poor QOL as compared to male patients. In this study, males have better QOL in social relationship domain as compared to females. The reason of better QOL in males is that male have better social relationships (strong relation and sexual activation) and support than females. These patients have more chances of outing and meeting friends which give them encouragement to face challenges of life. Similar observation was made by Santos PR et al.^16^ and Salim K et al.^16^Age is one of the important predictor of QOL of HD patients.^17^ According to Liu WJ et al,^18^ age more than forty years was a significant risk factor of QOL of HD patients. In this study, Age have negative relationship with physical and psychological health domain. As age increases QOL impairs. But it is different as compared to other study by Khaled Abdel-Kader et al.^19^ These findings are consistent with the longitudinal HRQOL data in the HD (HEMO) Study, and the North Thames Study findings^20^ suggest that targeting future interventions at younger patients with CKD may have a larger impact on improving HRQOL. Marital status affects QOL. The major reason for this is that unmarried persons are dependent on their families as compared to married persons who have to run whole family which increases the financial stress and finally affect QOL. In this study as majority of the patients are unemployed 103 (82.4%) so unemployment further increases the mental stress and effect QOL.

There is positive relationship between the level of school education and the QOL.^21^ Education level does not affect QOL of HD patients in overall analysis but literate patients have better QOL in Domain 1(Physical health) than Illiterate. The patients who are literate, they have better understanding of the disease and awareness regarding it treatment options. Patients who are satisfied with treatment and they accept it they have better working capacity and sleep and rest which improves their QOL. Eighteen patients were employed among 84(67.2%) literate patients whereas only four patents were employed in 41(32.8%) illiterate patients. This thing shows that with improvement in education, job opportunities are more which improves financial status and improves QOL in HD patients. Similar observation is made by Patti F et al.^22^ Employment has been found to be a vital factor improving the QOL of ESRD patients. In this study only 22(17.6%) patients are employed. Employment is the only factor which affects the other three domains amongst all four domains of QOL. According to Sathvik BS et al.,^23^ employment also affect three domains of QOL. Patients who are employed have better QOL in physical & psychological health and social relationship domain than unemployed patients. Similar observation is made by studies in Taiwan^24^ and Brazil.^25^ According to Bohlke M et al.^25^, there were only 11(8%) patients who were employed and having job, amongst them employment was a better predictor of QOL of HD patients. Employed patients can perform their jobs, have better body image, appearance and self esteem which improves their QOL than unemployed.^25^^,^^26^

Financial status effect QOL of HD patients. According to a report by Nadia Ayub and Zahid Iqbal, income has a positive influence on life satisfaction.^26^ Patients with better income level have no financial stress in getting a dialysis than in patient with lower income because they have good means of transport in case of medical emergency, better living, noise & pollution free environment and social activities which improve their QOL. Patients with good income support have more chances of availing opportunities for recreation and leisure activities which give them feeling of healthy life and improved QOL. Similar pattern is also observed by Seica A et al.^27^ According to him, lower socioeconomic status affect QOL in HD patients. Place of residence does not affect QOL in overall analysis but in domain wise analysis patients of rural areas in physical health and environment domain has better QOL as compared to patient living in Urban areas. Although there is improvement in living in urban areas and facilities are more as compared to rural areas but still QOL is better in rural areas. In urban areas, there is burden of traffic on the roads which hinders mobility. In urban areas, life is close to nature, environment is pollution free which improves QOL.

Total time consumed in getting HD effects QOL significantly in social relationship domain than other domains. Actually patients with good social relations were getting support in the form of private transport which helped them in getting HD timely. Moist LM et al,^28^ reported similar results. According to him, longer travel time was associated with lower QOL. Government of Pakistan has provided dialysis services at the doorstep of ESRD patients in THQ's and DHQ's hospitals.

Mode of transport effect QOL in environment domain than all other domains. Patients with private transport were having better QOL than public and other transports. Private transport provide them freedom for movement, physical safety and less exposure to physical environment like pollution, noise, traffic, climate and clumsy environment. In public transport they have to wait a lot at bus stops and they spent more time in getting HD. Distance covered to reach hospital effect QOL. Patients who were coming from distance more than 5km their QOL was impaired in psychological domain than other domains. Patients who were coming for more distances were having more worries in getting HD and they have to travel a lot in getting HD which increase the time. As already discussed, time consumed in getting HD effect QOL, perhaps underlying factor is distance covered to reach hospital and private transport.

## Limitation of the study:

1. Sample size is small in this study and need to enhance the number of the patients.
2. Patients from other major cities may also be added.
3. There is need to add clinical parameters like hemoglobin, hyperparathyroidism and adequacy of dialysis which also affects QOL of dialysis patients.
4. There is need to conduct prospective study to compare the QOL of the patients.

## CONCLUSION

Different factors like gender, age, marital status, unemployment, socioeconomic status, residence of rural area, distance covered to reach hospital, mode of transport, total time consumed in getting HD affect QOL of HD patient. Education level is a positive factor for improving QOL of HD patients.

## Authors contribution:

The concept of the research problem and final write up was completed by **Dr. Muhamamd Anees,** Literature review was done by **Mr. Muzamamil Riaz Malik, Mr. Tanzeel Abbasi **developed the questionnaire. Data collection was done by **Dr. Zeesahan Nasir,** data was entered by **Dr. Yasir Hussain,** whereas data was analysed by **Mr. Muhamamd Ibrahim.**
